# Adequate vitamin D level associated with reduced risk of sporadic colorectal cancer

**DOI:** 10.3389/fnut.2023.1024849

**Published:** 2023-01-26

**Authors:** Yanhui Ma, Lin Deng, Yuchan Huangfu, Yunlan Zhou, Ping Wang, Lisong Shen

**Affiliations:** Department of Laboratory Medicine, Xinhua Hospital, Shanghai Jiao Tong University School of Medicine, Shanghai, China

**Keywords:** vitamin D, 25(OH)D, sporadic colorectal cancer, risk factor, nutrition

## Abstract

**Purpose:**

The effect of vitamin D level pertinent to colorectal cancer incidence, progression, or mortality risk is complicated, and study findings are mixed. Therefore, we evaluated whether serum vitamin D [25-hydroxyvitamin D, 25(OH)D] is associated with the incidence of sporadic colorectal cancer (CRC).

**Methods:**

This study is a retrospective analysis of the relationship between serum 25(OH)D level and the risk of CRC. Age, sex, body mass index, history of polyp, disease conditions (i.e., diabetes), medications, and other eight vitamins were used as confounding factors. A total of 389 participants were enrolled in this study, including comprising 83 CRC patients without a family history and 306 healthy controls, between January 2020 and March 2021 at the Department of Colorectal Surgery and Endoscope Center at the Xinhua Hospital, Shanghai Jiao Tong University School of Medicine. Adjusted smoothing spline plots, subgroup analysis, and multivariate logistic regression analysis were conducted to estimate the relative risk between serum 25(OH)D and sporadic CRC risk.

**Results:**

After fully adjusting the confounding factors, it was found that circulating 25(OH)D played a protective role in patients with CRC (OR = 0.76 [0.63, 0.92], *p* = 0.004) and that an adequate vitamin D level was significantly associated with a reduced CRC risk compared to vitamin D deficiency or sufficiency (OR = 0.31 [0.11, 0.9], *p* = 0.03). According to this study, statins did not affect the potential protective effects of vitamin D (OR = 1.02 [0.97, 1.08], *p* = 0.44) and may account for the inverse association between serum 25(OH)D and colorectal cancer.

**Conclusion:**

An adequate level of serum 25(OH)D was associated with a reduced CRC risk, especially for the elderly. The finding on the absence of protective effect of vitamin D in the statin use subgroup, suggests it may be one of the substantial contributing confounders, and warrants further investigation.

## 1. Introduction

Colorectal cancer (CRC) is the third most common cancer in men and the second in women worldwide (1), while most CRC cases are sporadic and non-inherited, which is influenced by the local gut environment and accumulation of mutations and epigenetic changes. In addition, genetic predisposition is a risk of CRC, and there are several other risk factors strongly associated with colorectal cancer, such as being overweight or obese, smoking, heavy alcohol use, being older, having a history of adenomatous polyps, and having type 2 diabetes (2). Nutrients such as vitamins are considered to play an important role in the development and prevention of colorectal cancer (3, 4). Vitamin D was highlighted as an important player in numerous diseases (5–8). Its anti-inflammatory, immunomodulatory, proapoptotic, and antiangiogenic effects on inhibiting carcinogenesis and curbing tumor growth are outstanding both *in vivo* and *in vitro* (9, 10). DINOMIT (including seven phases: disjunction, initiation, natural selection, overgrowth, metastasis, involution, and transition), a new model of cancer pathogenesis, is strongly linked to vitamin D deficiency (11).

However, the evidence on whether vitamin D intake or serum levels affect cancer incidence, progression, or mortality is mixed in observational studies and clinical trials. A large number of studies reported that higher intake or circulating vitamin D was associated with a decreased CRC risk (12–14). Conversely, these are null findings from randomized trials and systematic reviews (15–18). In the most recent meta-analysis of RCTs including 10 randomized clinical trials (the Vitamin D and Omega-3 Trial [VITAL] trial), results demonstrated that vitamin D supplementation does not affect cancer incidence but does significantly reduce total cancer mortality rates up to 13% (19). In some cases, the absence of vitamin D effects could be due to incompletely following guidelines for designing and analyzing nutritional clinical studies (20), or maybe driven by unknown confounders. In addition, few studies are focusing on the joint effects of multiple vitamin co-exposure in CRC. One prospective cohort study found the associations between circulating concentrations of six common vitamins (viz., VA, VD, VE, VC, VB12, and VB9) and all-cause and cause-specific mortality risks depending on different exposure patterns (21). A growing body of evidence indicated that vitamin D might exert its biological functions in concert with drugs that are linked with vitamin D absorption and/or metabolism pathway like statins (22). After multivariable adjustment, whether increasing levels of vitamin D were associated with reduced risk of CRC is worth studying.

Herein, we aim to figure out the substantial relationship between serum 25(OH)D and CRC risk in the Chinese population. Furthermore, we explored whether the association varies according to several CRC confounding factors, including sex, age, lifestyle, multivitamin status, medication, and polyp history. This allows us to better understand the experimental and observational results on the association between circulation 25(OH)D and a low risk of CRC.

## 2. Materials and methods

### 2.1. Subject recruitment

This retrospective analysis of serum 25(OH)D enrolled 83 CRC cases and 306 controls who underwent endoscopy procedures for disease screening and/or the periodic health examination between January 2020 and March 2021 from the Department of Colorectal Surgery and Endoscope Center at the Xinhua Hospital, Shanghai Jiao Tong University School of Medicine (Shanghai, China). All the participants were continuously enrolled for over 1 year. According to their medical records, 83 CRC cases who underwent radical resection with histopathologic or cytologic specimens available were confirmed by one or two pathologists. Other 306 subjects without any other cancer history, such as breast cancer and lung cancer, autoimmune disease, inflammatory bowel disease, severe infectious diseases, and a BMI < 30 are defined as controls. Controls were randomly selected with respect to age. Clinical and laboratory characteristics of patients with CRC and controls are presented in Supplementary Table 1. Demographic characteristics and other vitamin results of patients with CRC and controls according to clinical cutoff concentrations of serum 25(OH)D are presented in Supplementary Tables 2, 3, respectively. Serological examination records before surgery and/or colonoscopy were chosen to analyze.

### 2.2. Serum samples and biochemical analyses

For the analyses, 5 ml of fasting blood from all participants was collected 1 or 2 days before surgery and/or colonoscopy. The peripheral blood was collected in a serum separator tube, and samples could clot for 30 min before centrifugation at 1000*g* for 5 min. All peripheral blood samples were processed within 2 h of collection. Serum thiamine (VB1), riboflavin (VB2), pyridoxine (VB6), folic acid (VB9), cobalamin (VB12), vitamin C, vitamin A, and vitamin E were analyzed by using an automatic electrochemistry analyzer (LK3000VI, Lanbiao, Tianjin, China) using commercially available kits. Serum 25(OH)D was measured using the Architect i2000 chemiluminescence immunoassay analyzer (Abbott, Illinois, USA). Inter-assay coefficients of variation (CV%) of vitamin D were less than 6%, and others were less than 10%.

### 2.3. Statistical analysis

Sample characteristics for the participants were compared using descriptive statistics and tested for significance using the Kruskal–Wallis rank sum test for continuous variables and proportions for categorical variables. If the variable number was less than 10, Fisher’s exact probability test was used. For logistic regression analysis, a minimum of 10 outcome events per predictor variable (EPV) is recommended. Considering the Chinese population, judgment criteria for vitamin D levels are as follows: serum 25(OH)D of <25 nmol/L is defined as vitamin D deficiency, 25(OH)D of 25–50 nmol/L is considered vitamin D insufficiency, 25(OH)D of 50–75 nmol/L is vitamin D adequate, and ≥75 nmol/L is defined as vitamin D sufficiency (23, 24). We applied a single-factor analysis of variables for CRC risk including sex, age, history of polyp, BMI, concomitant diseases (diabetes and hypertension), medications (aspirin, statins, and ACEI), and serum vitamin (VB1, VB2, VB6, VB9, VB12, vitamin C, vitamin A, and vitamin E) as confounding factors. Adjusted smoothing spline plots of serum 25(OH)D by mixed factors were created to study the shape of the relationship of 25(OH)D with risk of CRC based on continuous 25(OH)D and 25(OH)D subgroups, respectively. A subgroup analysis examined the association between 25(OH)D and CRC risk according to sex, history of polyp, BMI, statin use, and age. A logistic regression model was used to test for interaction and compare the odds ratio (OR) and 95% confidence interval (CI) among the analyzed subgroups. Multivariable logistic regression models were used to investigate the effects of 25(OH)D and the other variables on the occurrence of CRC. The multivariable regression model adjusted for factors including age (<45, 45–59, 60–74, and ≥75), polyp history (yes or no), diabetes (yes or no), hypertension (yes or no), currently smoking (yes or no), alcohol (yes or no), aspirin use (yes or no), statin use (yes or no), ACEI use (yes or no), BMI (<18.5, 18.5–24.9, and 25–30), and other eight vitamins. The association analyses were performed in continuous 25(OH)D per 10 nmol/L and 25(OH)D groups, respectively. Stratified analyses and assessment of statistical interaction on the multiplicative scale were also performed in 25(OH)D sub-cohorts defined by sex, history of polyp, BMI, statin use, and age. All statistics were two-tailed, and a *P*-value of <0.05 was considered statistically significant. All data were analyzed and visualized using multiple R packages *via* The R Foundation^1^ or SPSS 26.0. In the R package mgcv, the gam function was used to fit the generalized additive model on each tile separately.

## 3. Results

**FIGURE 1:**
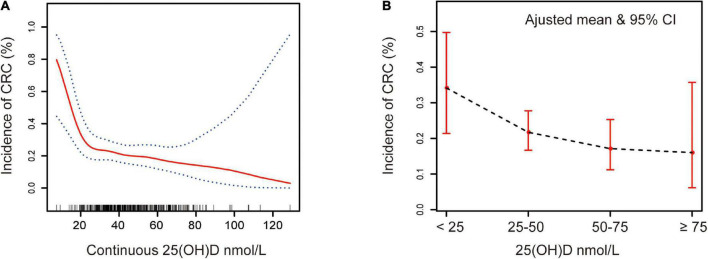
Association between serum 25(OH)D and CRC. Adjusted for sex, age, polyp history, diabetes, hypertension, smoking, alcohol, aspirin, statins, ACEI, VB1, VB2, VB6, VB9, VB12, VC, VE, VA, and BMI. **(A)** Red solid line represents the spline plots of continuous 25(OH)D concentration, and the blue dotted line represent the 95% confidence intervals of the spline plots. **(B)** The bar plot of four-level serum 25(OH)D groups with adjusted mean and 95% CIs. Vitamin D deficiency: 25(OH)D < 25 nmol/L, vitamin D insufficiency: 25(OH)D 25–50 nmol/L, vitamin D adequate: 25(OH)D 50–75 nmol/L, and vitamin D sufficiency: 25(OH)D ≥ 75 nmol/L.

In this retrospective analysis, participants were classified into four categories, namely, deficient, insufficient, adequate, and sufficient, according to the serum 25(OH)D level, as described in the Section “2. Materials and methods.” Supplementary Figure 1 is a flowchart for participant recruitment. Table 1 describes the characteristics of the subjects, including demographic characteristics and other vitamin results. Individuals with higher serum 25(OH)D were less likely to have tumors than those with low serum 25(OH)D (16 vs. 34%, *p* = 0.13). Figure 1A presents an overall smoothing spline plot of continuous 25(OH)D and the risk of CRC. As serum 25(OH)D concentration increased, the incidence of CRC significantly declined. The reanalysis result was consistent when data were modified into four groups: vitamin D deficiency, insufficiency, adequate, and sufficiency (Figure 1B).

**TABLE 1:** Characteristics of the study participants according to clinical cutoff concentrations of serum 25(OH)D.

Clinical cutoffs		25(OH)D (nmol/L)			*P*-value
	<25	25–50	50–75	≥75	
No. of participants (%)	41 (11%)	212 (55%)	111 (29%)	25 (6.4%)	
CRC, N (%)	14 (34%)	46 (22%)	19 (17%)	4 (16%)	0.13
Age	66 ± 17	63 ± 13	62 ± 11	63 ± 11	0.40
Sex					0.30
Female	20 (49%)	91 (43%)	38 (34%)	9 (36%)	
Male	21 (51%)	121 (57%)	73 (66%)	16 (64%)	
BMI	22 ± 2.7	23 ± 2.7	23 ± 2.7	23 ± 2.5	0.04
**Vitamins**					
VB1 (nmol/mL)	59 ± 13	58 ± 8	61 ± 9	60 ± 9	0.03
VB2 (ng/mL)	241 ± 45	240 ± 41	240 ± 41	247 ± 37	0.87
VB6 (μmol/mL)	20 ± 4.8	20 ± 5.0	20 ± 5.3	20 ± 4.3	0.82
VB9 (nmol/mL)	14 ± 6.3	13 ± 5.5	14 ± 5.5	15 ± 6.1	0.31
VB12 (pg/mL)	269 ± 125	272 ± 99	271 ± 83	282 ± 107	0.96
Vitamin A (μmol/mL)	0.71 ± 0.22	0.80 ± 0.24	0.83 ± 0.24	0.91 ± 0.27	0.005
Vitamin C (μmol/mL)	43 ± 7.4	39 ± 5.2	40 ± 4.9	39 ± 4.1	<0.001
Vitamin E (μg/mL)	11 ± 1.0	11 ± 1.0	12 ± 1.2	11 ± 1.2	0.001
**Diseases and medications**					
Polyp history	4 (9.8%)	40 (19%)	27 (24%)	7 (28%)	0.16
Diabetes	5 (12%)	42 (20%)	21 (19%)	3 (12%)	0.56
Hypertension	28 (68%)	115 (54%)	50 (45%)	11 (44%)	0.06
Smoke	4 (9.8%)	22 (10%)	20 (1%)	4 (16%)	0.22
Alcohol	2 (4.9%)	17 (8.0%)	14 (13%)	3 (12%)	0.39
Aspirins	9 (22%)	29 (14%)	6 (5.4%)	2 (8%)	0.02
Statins	4 (9.8%)	27 (13%)	11 (9.9%)	3 (12%)	0.87
ACEI	6 (14.6%)	4 (1. 9%)	3 (2.7%)	1 (4%)	<0.001

BMI, body mass index; ACEI, angiotensin converting enzyme inhibitors.

Values are means (SD) or numbers (%), otherwise specified.

**FIGURE 2:**
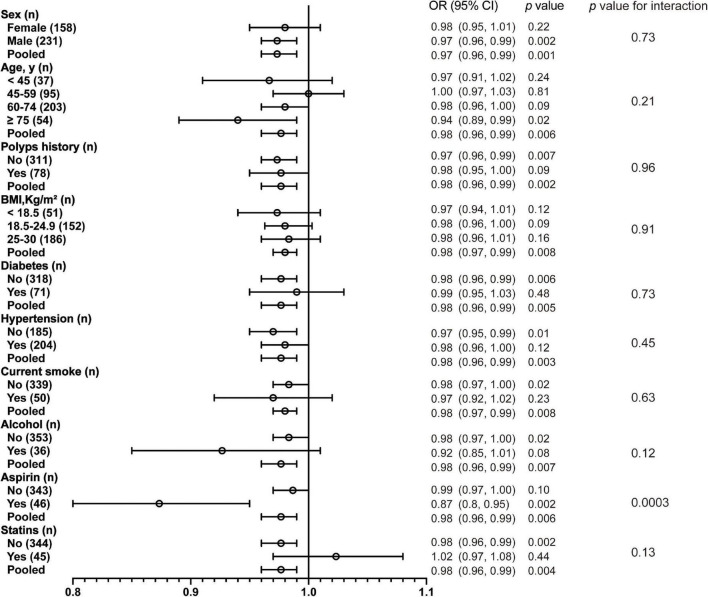
Association between 25(OH)D per 10 nmol/L and CRC according to baseline characteristics. BMI, body mass index.

**FIGURE 3:**
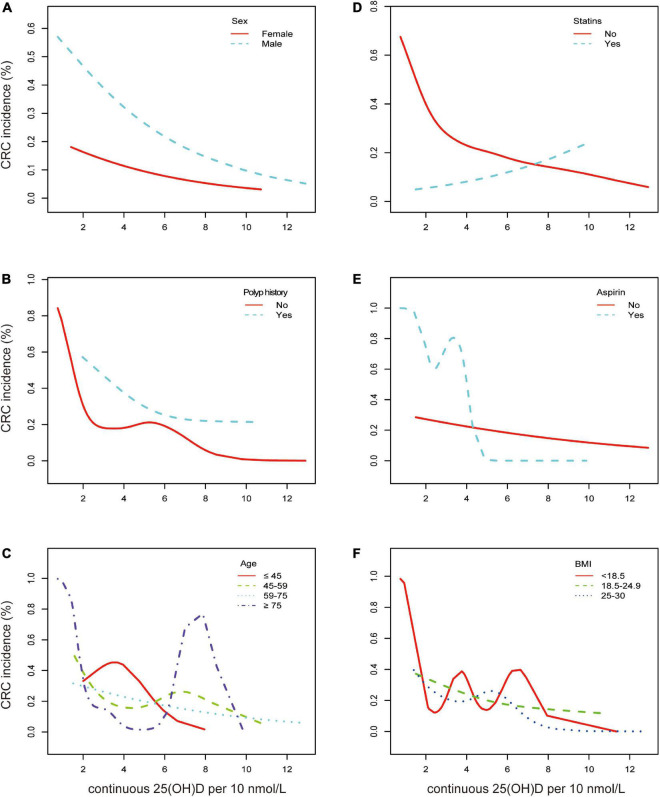
Association between continuous 25(OH)D per 10 nmol/L and CRC according to baseline characteristics. Smooth fitting curve adjusted for sex, age, polyps history, diabetes, hypertension, smoking, alcohol, aspirin, statins, ACEI, VA, VB1, VB6, VB9, VB12, VC, VE, VD, and BMI. **(A)** Aqua dashed line represents the spline plots of men and the red solid line represents those of women. **(B)** Aqua dashed line represents the spline plots of polyp history and red solid line represents participants without polyp. **(C)** Red solid line represents the spline plots of age < 45 years, green dashed line represents age between 45 and 59 years, aqua dotted line represents age between 60 and 75 years, and the purple dash-dotted line represents age ≥ 75 years. **(D)** Aqua dashed line represents the spline plots of statin treatment and red solid line represents participants without statin treatment. **(E)** Aqua dashed line represents the spline plots of aspirin use and red solid line represents participants without aspirin. **(F)** Red solid line represents the spline plots of BMI < 18.5, green dashed line represents BMI between 18.5 and 24.9, and blue dotted line represents BMI between 25 and 30.

As a result of a single-factor analysis of CRC risk based on continuous 25(OH)D per 10 nmol/L, several factors, such as sex, age (<45, 45–59, 60–74, and ≥75), BMI (<18.5, 18.5–24.9, and 25–30), polyp history, and medications, were identified to have a strong relationship with CRC (Supplementary Table 4). As another step, we conducted a separate regression analysis using continuous 25(OH)D values stratified by sex, age, polyp history, BMI, concomitant diseases, smoking, alcohol, statin, and aspirin use. The associations of these exposures with CRC are presented in Figure 2 and Supplementary Table 5. After stratification by all confounding factors, the risk of CRC was decreased in all subgroups with elevated 25(OH)D, other than those who used statins (Figure 3; Supplementary Figure 2). There was no significant heterogeneity among analyzed subgroups based on sex, age, polyp history, BMI, concomitant diseases, smoking, alcohol consumption, and statin use, except for aspirin use.

It was noteworthy that our results indicated that 25(OH)D did not act as a protective factor but promoted the risk of CRC in participants receiving statins (OR = 1.02 [0.97, 1.08], *p* = 0.44) (Figure 3D). The remaining data showed that men had a much higher risk of CRC, and vitamin D had a stronger protective effect on men (OR = 0.97 vs. 0.98, *p* = 0.002) (Figure 3A). It was found that participants with a polyp history were more likely to report CRC, and the risk declined with higher serum vitamin D levels (Figure 3B). In the elderly (over 75 years old), vitamin D could have a significant effect on the prevalence of CRC (OR = 0.94 [0.89, 0.99], *p* = 0.02) as the CRC incidence was depicted as a U shape. In the other three age subgroups, the incidence decreased likewise (Figure 3C). Participants under aspirin medication at a low range of serum 25(OH)D were more likely to have CRC (Figure 3E). Consistent trends between 25(OH)D and CRC incidence in BMI categories, diabetes, hypertension, smoking, and alcohol consumption were found (Supplementary Figure 2).

Furthermore, a multivariate regression analysis was used to investigate the effects of serum 25(OH)D on CRC incidence (Table 2). When confounding factors are not taken into account, 25(OH)D can lead to a 40–50% reduction in CRC risk. After fully adjusting the confounding factors that may influence CRC occurrence, an adequate level of 25(OH)D is protective against CRC, based on subdivided vitamin D categories (OR = 0.31[0.11, 0.92], *p* = 0.03).

**TABLE 2:** Individual effect of serum 25(OH)D concentrations on CRC.

Exposure	Incidence, n (%)	Non-adjusted		Adjust I		Adjust II		Adjust III	
		OR (95% CI)	*P*-value	OR (95% CI)	*P*-value	OR (95% CI)	*P*-value	OR (95% CI)	*P*-value
Continuous 25(OH)D per 10 nmol/L	83 (21%)	0.8 (0.7, 0.9)	0.005	0.8 (0.7, 0.9)	0.002	0.8 (0.6, 0.9)	0.004	0.76 (0.63, 0.92)	0.004
**Clinical cutoffs**									
<25 nmol/L	14 (34%)	Reference		Reference		Reference		Reference	
25–50 nmol/L	46 (22%)	0.5 (0.3, 1.1)	0.09	0.5 (0.2, 1.1)	0.09	0.5 (0.2, 1.2)	0.13	0.56 (0.22, 1.42)	0.22
50–75 nmol/L	19 (17%)	0.4 (0.2, 0.9)	0.03	0.3 (0.1, 0.8)	0.02	0.3 (0.1, 0.9)	0.03	0.31 (0.11, 0.92)	0.03
≥75 nmol/L	4 (16%)	0.4 (0.1, 1.3)	0.12	0.3 (0.1, 1.2)	0.09	0.3 (0.1, 1.2)	0.10	0.32 (0.07, 1.42)	0.14

Adjust I model was adjusted for sex, BMI grade, and age grade.

Adjust II model was adjusted for sex, BMI grade, age grade, polyp history, diabetes, hypertension, smoking, alcohol, aspirin, statins, and ACEI.

Adjust III model was adjusted for sex, BMI grade, age grade, polyp history, diabetes, hypertension, smoking, alcohol, aspirin, statins, ACEI, VB1, VB2, VB6, VB9, VB12, VC, VA, and VE.

## 4. Discussion

There is strong evidence that supports the UVB–vitamin D–cancer hypothesis that arose from an inspection of the geographic distribution of colon cancer deaths in the United States (25). In this study, we found that an adequate serum 25(OH)D level protected against sporadic CRC in the Chinese population and that this association was significantly modified by major factors, i.e., age, sex, polyp history, concomitant diseases, currently smoking, medications, BMI, and other vitamins. In addition, no significant interaction was found across each factor, except for aspirin. Furthermore, our results suggested that vitamin D was not a protective factor for the subgroup of statins and may contribute to an increased risk of CRC, which requires further investigation.

Several factors contribute to the pathogenesis of sporadic CRC, including genetics and environment, as well as diet and malfunctional gut microbiota, which is regarded as a key point (26, 27). Nutrients and foods also may cooperate, as a dietary pattern, to influence colorectal cancer risk. Rather than nutrition, vitamin D was considered to be a hormone. It may directly or indirectly mediate 3–5% of the human gene expression and had been confirmed in a wide spectrum of anticancer activities: anti-proliferation, induction of differentiation and apoptosis, anti-inflammation, inhibition of invasion and metastasis, and suppression of angiogenesis in experimental studies (9, 28).

It appeared that vitamin D has an inconsistent and intricate association with CRC risk. Low levels of 25(OH)D have been associated with increased cancer incidence and mortality in several observational studies (21, 29). In a meta-analysis of 16 prospective cohort studies with a large population (30), a 50 nmol/L (20 ng/mL) increase in 25(OH)D levels led to a reduction of 11% in cancer incidence rates and a 24% reduction in cancer mortality rates in women. Another meta-analysis of eight prospective studies on the association between serum 25(OH)D levels and cancer incidence and mortality found that cancer risk decreased by 7% and cancer mortality rates decreased by 2% with each increase in serum 25(OH)D levels of 20 nmol/L (8 ng/mL) (31). A Japanese prospective study found higher vitamin D concentration was associated with a lower risk of total cancer (32). The findings of these studies are consistent with the notion that vitamin D may have protective effects against cancer, but not all observational studies showed an association between higher vitamin D status and cancer prevention. There are null findings between vitamin D and CRC risk as well. A systematic review plus meta-analysis did not find evidence to suggest that vitamin D supplementation alone reduces the incidence of cancer or cancer mortality, even after including long-term follow-up results (15). A randomized, placebo-controlled trial found supplementation with vitamin D did not result in a lower incidence of invasive cancer than a placebo (17). Cumulating evidence reported a U-shaped effect of vitamin D on the risk relationship with diseases (33, 34). For standardization of serum total of 25(OH)D values above 100 nmol/L, a higher value than we observed, the risks did not continue to decrease (33). In this study, our results agreed with the conclusion that an adequate level, not the highest vitamin D concentration, led to the most beneficial outcome. However, the optimal serum 25(OH)D for colorectal cancer for the population of Asia may be different from international public health recommendations. Our findings suggested an adequate level of vitamin D (50–75 nmol/L) was preferred according to a Chinese consensus on bone health. Therefore, solid evidence from large population studies is needed to relate to determining nutrient recommendations.

It is important to understand the physiology of vitamin D because about half of the population is diagnosed with deficiency following clinical guidelines based on observational studies. Sources of vitamin D hormones included both endogenous sources, i.e., ultraviolet light, and exogenous sources, i.e., certain foods and dietary supplements. For both sources, the D3 carried in the bloodstream on either DBP or lipoproteins underwent a two-step sequential hydroxylation (25-hydroxylase and 1-alpha–hydroxylase) to produce an active metabolite, 1,25(OH)2D3 (also referred to as calciferol) (35). For dietary vitamin D3, it depended on the cytochrome P450 enzyme CYP27B1 polymorphism as well (36). Downstream vitamin D functioned by binding to and activating the nuclear VDR. A randomized clinical trial demonstrated that the effectiveness of vitamin D3 supplementation on advanced adenomas, but not on adenoma, varied according to genotype at two VDR SNPs (rs7968585 and rs731236). For rs7968585 with the AA genotype, vitamin D3 supplementation reduced risk by 64%. While for G or GG alleles, vitamin D3 supplementation increased the risk by 41% (37). In the meantime, the extrarenal production of 1,25(OH)2D3, likely for paracrine or autocrine uses, was recognized wildly in many tissues, including the epidermis and other epithelial tissues, bone, placenta, and tumors (38). The main reason was that CYP27B1 expression was induced in these extrarenal cells like colorectal carcinoma cells (39). However, the enzyme CYP24A1 or 24-hydroxylase, which degraded 1,25(OH)2D3 to inactive calcitroic acid, was reported upregulated in tumor cells or other cells. Some studies might not find any 1,25 (OH)2D3 effects after longer follow-up periods due to its degradation over time (10, 40). Meta-analyses of cancer incidence with respect to dietary intake had limited success (10). The effect of vitamin D supplementation at a given dose was presumably based on the baseline 25(OH)D level of the study population and the achieved vitamin D status after treatment (34). Clinical guidelines recommend measuring circulating 25(OH)D as a marker of vitamin D deficiency (41). Therefore, we accessed the association between circulating 25(OH)D and CRC risk rather than dietary vitamin D intake.

Over the past several decades, chemoprevention has been extensively studied as a strategy for reducing the risk of CRC. Aspirin has the strongest evidence that it can lower the risk of CRC (42). Although studies of aspirin prevention of CRC have produced mixed results, reporting both significant (43) and non-significant results (44), most evidence supported an association with decreased risk of CRC (45). In addition, the Aspirin in Reducing Events in Elderly (ASPREE) trial found that aspirin use increased mortality due to all causes and cancer-related mortality, as well as CRC risk (HR 1.77, CI [1.02–3.06]) (46). There were few studies on drug interactions between vitamin D and aspirin. Herein, in the aspirin subgroup, our results supported the beneficial role of aspirin and high-level vitamin D. We still have to work on knowledge gaps such as molecular mechanisms and the application of genomic tools to understand interaction better.

Statin is another important chemoprevention agent that is usually applied to lower cholesterol for the primary prevention of cardiovascular disease (CVD) events. Vitamin D supplements may interact with statin medications because cholecalciferol, the endogenous vitamin D precursor, is derived from cholesterol. Statins may reduce vitamin D synthesis downstream and increase the concentration of vitamin D in the blood (22, 47). Statins and vitamin D appear to compete for the same metabolizing Cytochrome P450 enzyme (48), so high intakes of vitamin D, especially from supplements, may reduce the potency of statins (47, 49). In addition, there is evidence that dysregulation of cholesterol and vitamin D metabolism is associated with age-related diseases (50). Here, we did not focus on the underlying mechanism between vitamin D and statins. But it is worth noting that the protective effect of vitamin D was absent in the statin use subgroup. Alternatively, it showed a significant association between statin use and increased risk of CRC. It supported the interaction and/or competition of vitamin D and statin metabolism existed and proposed an explanation for inconsistent results produced by epidemiological and clinical studies of statins and colorectal neoplasia.

Studies have suggested that a higher BMI greatly raises CRC risk among both men and women (51, 52). In our study, primary data analysis indicated an association between BMI and CRC risk, and serum vitamin D protected against CRC when BMI was ≤30 (data not shown). This assumed certain confounding factors existed, such as adiposity-associated metabolites, genetic variants, etc. The main VITAL study showed a possible reduction of total cancer incidence for individuals with normal BMI, but not for individuals with overweight or obesity (18). Body fat distribution and impaired adipose tissue function, rather than BMI, might be better indicators of risk. As reported that polyps were highly associated with CRC (53, 54), we also found that individuals with no polyp history and higher serum 25(OH)D appeared to be at lower risk for CRC.

Older adults were at increased risk of developing vitamin D deficiency, and vitamin D metabolism became impaired. Age-related decline in kidney function associated with progressive structural deterioration of the kidney could affect vitamin D metabolism, leading to the suppression of 1,25(OH)2D3 synthesis (55). One cohort research showed a higher dietary intake of vitamin D was associated with a reduced CRC risk in older adults in the framework of the PREDIMED cohort (12). As previously reported, among randomized VITAL participants (mean [SD] age: 67.1 [7.1] years), no significant differences in cancer incidence by vitamin D treatment were observed (18). We found a U-shaped correlation between serum 25(OH)D and the incidence of CRC in the elderly subgroup, indicating that an adequate level of vitamin D was more important for the elderly. Despite this, we should exercise caution when using high doses of vitamin D in older (19). Overall, we need a population-based study to explore the effect of each exposure of CRC incidence or recurrence.

Vitamin D plays a critical role in calcium and phosphate metabolism as well. A 4-year randomized clinical trial showed that supplementing with vitamin D3 and calcium did not significantly reduce all-type cancer risk among healthy postmenopausal women (56). Studies found that healthy women who took vitamin D and calcium supplements for an average of 7 years did not have a reduced incidence of colorectal cancer (57). A clinical trial based on an ancillary study of data from the VITAL trial revealed that colorectal adenomas or serrated polyps did not appear linked to vitamin D supplementation, but calcium and vitamin D together almost quadruple the risk (58). Moreover, as we all know, vitamins cannot be consumed separately in a daily diet. A growing body of evidence suggests that vitamin interactions are related to diseases (21). We are aware of fewer studies taking any single vitamin as a confounder factor to access the relationship between vitamin D and CRC risk. In view of the interaction with other metabolites like calcium and statins, vitamin D analogs were developed. Chemicals with similar structures and anticancer properties to vitamin D, but with fewer potential side effects (59).

In general, the population of our study for factors affecting CRC incidence was relatively small; therefore, a large population-based study would be needed to investigate the effects of each exposure on CRC. There are also several potential limitations in this retrospective study. First, as a retrospective analysis of observational studies with a limited population, we cannot rule out another residual or unknown confounding as a potential explanation for the observed findings. Nevertheless, we conducted consistent results based on two modeling data [continuous 25(OH)D per 10 ng/mL and 25(OH)D subdivided groups] that overall higher serum 25(OH)D was inversely related to sporadic CRC. Second, cohort studies are less susceptible to selective bias compared with case–control studies. As for case–control studies, the selection of cases may not be representative of all cases within the population. Herein, we obtained laboratory and clinical data of cases and controls before clinical diagnosis, which would be less susceptible to selective bias. Third, our study only performed one single serum 25(OH)D assessment before surgery. This assessment would be compromised if any changes occurred such as dietary habits influenced by diseases. Thus, we applied appropriate inclusion criteria and conducted subgroup and sensitivity analysis, we did not find serum 25(OH)D had significant interaction with other major exposures except for aspirin use.

## 5. Conclusion

This retrospective study indicated that an adequate serum 25(OH)D is associated with a lower CRC risk. The association between vitamin D and CRC risk was modified by sex, age, polyp history, disease conditions, medications, BMI, and other CRC-related vitamin levels. Our findings supported the hypothesis that vitamin D may grant protection against the risk of cancer. Nonetheless, the lower risk associated with higher circulating vitamin D concentration seemed to show a U-shaped effect, suggesting the highest 25(OH)D level may not provide optimal benefits, especially for the elderly (≥75 years old). The role of serum 25(OH)D and its interaction with other nutrition, genetic variants, cancer subsites, prognosis, and mortality on CRC should be subject to further studies.

## Data availability statement

The datasets presented in this article are not readily available for ethical reasons. Requests to access the datasets should be directed to the corresponding authors.

## Ethics statement

This present study conformed to the principles of the Declaration of Helsinki. Approval was obtained from the Research Ethics Committee of the Xinhua Hospital Affiliated to Jiao Tong University School of Medicine. The patients/participants provided their written informed consent to participate in this study.

## Author contributions

YM designed the study and performed the statistical analysis. YM, LD, and YH collected the data. YM and YZ wrote the manuscript. PW and LS critically revised the manuscript. All authors contributed to the data interpretation and edited, reviewed, and approved the final manuscript.

## Footnotes

1
http://www.r-project.org; version 3.4.3.
