# Supplementary material for: Adequate vitamin D level associated with reduced risk of sporadic colorectal cancer
Source: Front Nutr. 2023 Jan 26;10:1024849. doi: 10.3389/fnut.2023.1024849 (PMC9908961; doi:10.3389/fnut.2023.1024849)
Supplement: Supplementary file 1 [file Data_Sheet_1.docx]

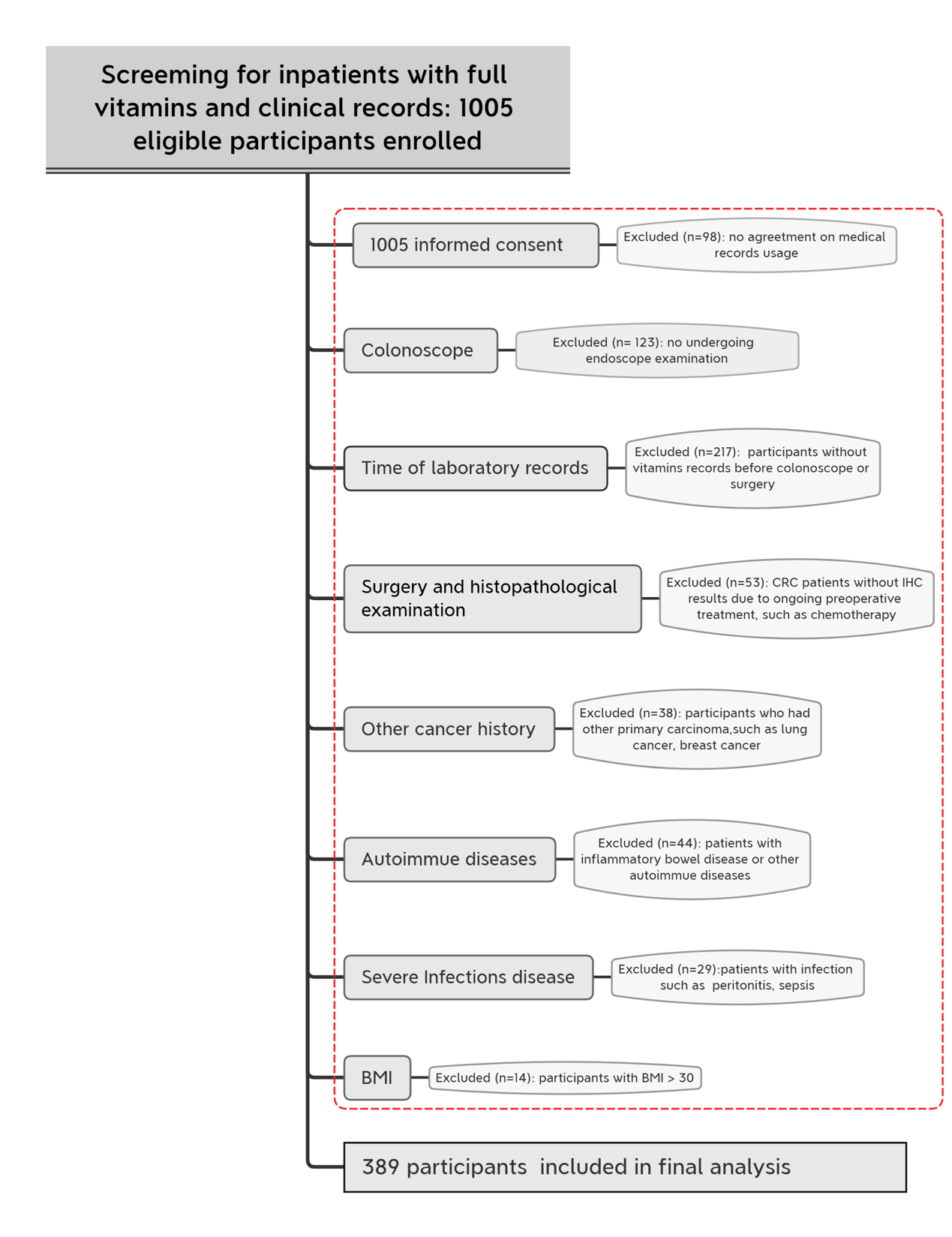


Supplementary figure.1 Participant Flow Chart

Supplementary Table. 1 Major clinical and laboratory characteristics of CRC patients and healthy controls

|  | CRC | HD | P-value |
| --- | --- | --- | --- |
| No. of participants | 83 | 306 |  |
| 25(OH)D (nmol/L) | 42 ± 16 | 48 ± 18 | 0.006 |
| Age | 60 ± 11 | 64 ± 11 | 0.08 |
| Male | 67 (81%) | 164 (55%) | <0.001 |
| BMI | 23 ± 3.0 | 23± 3.0 | 0.31 |
| Lifestyle |  |  |  |
| Smoke | 6 (7.2%) | 44 (14%) | 0.86 |
| Alcohol | 5 (6.0%) | 31 (10%) | 0.999 |
| Aspirins | 7 (8.4%) | 39 (13%) | 0.99 |
| Statins | 7 (8.4%) | 38 (12%) | 0.99 |
| Diabetes | 10 (12%) | 61 (20%) | 0.81 |
| Hypertension | 45 (54%) | 159 (52%) | 0.72 |
| Vitamins |  |  |  |
| VB1 (nmol/mL) | 59 ± 9.2 | 60 ± 7.7 | 0.40 |
| VB2 (ng/mL) | 253 ± 47 | 238 ± 40 | 0.007 |
| VB6 (μmol/mL) | 20± 5.0 | 21 ± 5.1 | 0.11 |
| VB9 (nmol/mL) | 13 ± 4.2 | 14 ± 6.0 | 0.47 |
| VB12 (pg/mL) | 250 ± 39 | 278 ± 106 | 0.61 |
| VA (μmol/mL) | 0.84± 0.25 | 0.81 ± 0.24 | 0.33 |
| VC (μmol/mL) | 40± 5.8 | 40 ± 5.0 | 0.42 |
| VE (ug/mL) | 11± 0.97 | 11± 1.1 | 0.41 |

The statistical evaluation was performed with GraphPad Prism (version 9.0). Values are shown as number (percentage), mean ± SD. A Student *t* test was used to analyze the differences between the groups for equal variances. Nonparmetric Kolmogorov-Smirnov test was used to analyze unequal variances data. Abbreviations: BMI, body mass index.

Supplementary Table. 2 Characteristics of 306 controls according to clinical cutoffs concentrations of serum 25(OH)D.

| clinical cutoffs |  |  | 25(OH)D (nmol/L) | |  |  | P-value |
| --- | --- | --- | --- | --- | --- | --- | --- |
|  |  | ＜25 | 25-50 | 50-75 | ≥75 |  |  |
| No. of participants (%) |  | 27 (7.8%) | 166 (53%) | 92 (31%) | 21 (7.2%) |  |  |
| 25(OH)D (nmol/L) |  | 21 ± 2.6 | 39 ± 6.5 | 61 ± 7.0 | 88 ± 14 |  | <0.001 |
| Age |  | 66 ± 15 | 64 ± 11 | 63 ± 10 | 64 ± 8 |  | 0.33 |
| Sex |  |  |  |  |  |  | 0.18 |
| Female |  | 16 (59%) | 82 (49%) | 35 (38%) | 9 (43%) |  |  |
| Male |  | 11 (41%) | 84 (51%) | 57 (62%) | 12 (57%) |  |  |
| BMI |  | 22 ± 2.9 | 23 ± 2.9 | 23 ± 3.0 | 23 ± 2.9 |  | 0.33 |
| Vitamins |  |  |  |  |  |  |  |
| VB1 (nmol/mL) |  | 56 ± 9.8 | 58 ± 8.6 | 61 ± 9.5 | 61 ±8.8 |  | 0.008 |
| VB2 (ng/mL) |  | 230 ± 40 | 240 ± 39 | 236 ± 40 | 248 ± 38 |  | 0.42 |
| VB6 (μmol/mL) |  | 19 ± 3.9 | 20 ± 5.4 | 19 ± 4.9 | 19 ± 4.1 |  | 0.62 |
| VB9 (nmol/mL) |  | 15 ± 7.0 | 13 ± 5.9 | 14 ± 5.6 | 15 ± 6.4 |  | 0.48 |
| VB12 (pg/mL) |  | 285 ± 151 | 278 ± 109 | 270 ± 79 | 302 ± 111 |  | 0.46 |
| Vitamin A (μmol/mL) |  | 0.69 ± 0.21 | 0.80 ± 0.24 | 0.83 ± 0.23 | 0.89 ± 0.27 |  | 0.02 |
| Vitamin C (μmol/mL) |  | 41 ± 6.2 | 39 ± 5.0 | 40 ± 4.7 | 40 ± 4.8 |  | 0.55 |
| Vitamin E (ug/mL) |  | 11 ± 1.1 | 11 ± 1.0 | 11 ± 1.1 | 11 ± 1.2 |  | 0.04 |
| Diseases and medications |  |  |  |  |  |  |  |
| Polyp history |  | 1 (3.7%) | 26 (16%) | 23 (25%) | 4 (19%) |  | 0.07 |
| Diabetes |  | 5 (19%) | 35 (21%) | 18 (20%) | 3 (14%) |  | 0.55 |
| Hypertension |  | 20 (74%) | 90 (54%) | 39 (42%) | 10 (48%) |  | 0.87 |
| Smoke |  | 3 (11%) | 20 (12%) | 18 (20%) | 3 (14%) |  | 0.17 |
| Alcohol |  | 1 (3.7%) | 15 (9.0 %) | 13 (14%) | 2 (9.5%) |  | 0.21 |
| Aspirins |  | 5 (11%) | 26 (16%) | 6 (6.5%) | 2 (9.5%) |  | 0.49 |
| Statins |  | 4 (14.5%) | 22 (13%) | 9 (9.8%) | 3 (14%) |  | 0.73 |

The statistical evaluation was performed with GraphPad Prism (version 9.0). Values are shown as number (percentage), mean ± SD. Ordinary one-way ANOVA was used to analyze the differences between the groups for equal variances. Nonparmetric Kruskal-Wallis test was used to analyze unequal variances data. Abbreviations: BMI, body mass index.

Supplementary Table. 3 Characteristics of 83 CRC according to clinical cutoffs concentrations of serum 25(OH)D.

| clinical cutoffs |  |  | 25(OH)D (nmol/L) | |  |  | P-value |
| --- | --- | --- | --- | --- | --- | --- | --- |
|  |  | ＜25 | 25-50 | 50-75 | ≥75 |  |  |
| No. of participants (%) |  | 14 (17%) | 46 (55%) | 19 (23%) | 4 (4.8%) |  |  |
| 25(OH)D (nmol/L) |  | 15 ± 6.0 | 36 ± 6.1 | 58 ± 7.2 | 88 ± 14 |  | <0.001 |
| Age |  | 66 ± 15 | 64 ± 11 | 63 ± 10 | 65 ± 8 |  | 0.055 |
| Sex |  |  |  |  |  |  | 0.61 |
| Female |  | 3 (29%) | 9 (20%) | 3 (16%) | 0 (0%) |  |  |
| Male |  | 10 (71%) | 37 (80%) | 16 (84%) | 4 (100%) |  |  |
| BMI |  | 21 ± 2.6 | 24 ± 3.1 | 23 ± 2.9 | 21 ± 1.0 |  | 0.12 |
| Vitamins |  |  |  |  |  |  |  |
| VB1 (nmol/mL) |  | 66 ± 15 | 60 ± 7.2 | 61 ± 7.3 | 58 ± 4.3 |  | 0.63 |
| VB2 (ng/mL) |  | 264 ± 43 | 251 ± 51 | 261 ± 39 | 263 ± 24 |  | 0.79 |
| VB6 (μmol/mL) |  | 23 ± 4.7 | 19 ± 4.1 | 22 ± 6.1 | 22 ± 3.8 |  | 0.03 |
| VB9 (nmol/mL) |  | 13 ± 4.3 | 13 ± 4.3 | 12 ± 3.3 | 15 ± 3.0 |  | 0.65 |
| VB12 (pg/mL) |  | 244 ± 36 | 247 ± 35 | 261 ± 44 | 243 ± 46 |  | 0.54 |
| Vitamin A (μmol/mL) |  | 0.71 ± 0.21 | 0.80 ± 0.23 | 0.91 ± 0.28 | 1.1 ± 0.10 |  | 0.03 |
| Vitamin C (μmol/mL) |  | 46 ± 8.8 | 39 ± 5.5 | 42 ± 5.1 | 37 ± 1.5 |  | 0.02 |
| Vitamin E (ug/mL) |  | 11 ± 0.58 | 11 ± 0.9 | 12 ± 1.1 | 11 ± 0.24 |  | 0.02 |
| Diseases and medications |  |  |  |  |  |  |  |
| Polyp history |  | 3 (21%) | 14 (33%) | 4 (21%) | 3 (75%) |  | 0.02 |
| Diabetes |  | 0 (0%) | 7 (15%) | 3 (16%) | 0 (0%) |  | 0.37 |
| Hypertension |  | 8 (57%) | 25 (54%) | 11 (58%) | 1 (25%) |  | 0.92 |
| Smoke |  | 1 (7.1%) | 2 (4.3%) | 2 (11%) | 1 (25%) |  | 0.88 |
| Alcohol |  | 1 (7.1%) | 2 (4.3%) | 1 (5.3%) | 1 (25%) |  | 0.96 |
| Aspirins |  | 4 (29%) | 3 (6.5%) | 0 (0%) | 0 (0%) |  | 0.06 |
| Statins |  | 0 (0%) | 5 (11%) | 2 (11%) | 0 (0%) |  | 0.83 |

The statistical evaluation was performed with GraphPad Prism (version 9.0). Values are shown as number (percentage), mean ± SD. Ordinary one-way ANOVA was used to analyze the differences between the groups for equal variances. Nonparmetric Kruskal-Wallis test was used to analyze unequal variances data. Abbreviations: BMI, body mass index.

Supplementary Table. 4 Single-factor analysis of variables for CRC risk.

| Nominal variables (n) | Descriptives | Single-factor analysis |  |
| --- | --- | --- | --- |
|  |  | OR (95% CI) | p value |
| continuous 25(OH)D (nmol/L) | 46 ± 18 | 0.98 (0.96, 0.99) | 0.005 |
| continuous 25(OH)D per 10 nmol/L | 4.6 ± 1.8 | 0.81 (0.70, 0.94) | 0.005 |
| 25(OH)D Clinical cutoffs |  |  |  |
| ＜25 nmol/L (41) | 41 (11%) | Reference |  |
| 25-50 nmol/L (212) | 212 (55%) | 0.53 (0.26, 1.10) | 0.09 |
| 50-75 nmol/L (111) | 111 (29%) | 0.40 (0.18, 0.90) | 0.03 |
| ≥75 nmol/L (25) | 25 (6.4%) | 0.37 (0.11, 1.28) | 0.12 |
| Sex |  |  |  |
| Female (158) | 158 (41%) | Reference |  |
| Male (231) | 231 (59%) | 3.32 (1.86, 5.92) | <0.0001 |
| Age (y, continuous) | 63 ± 13 | 0.99 (0.97, 1.01) | 0.33 |
| Age grade |  |  |  |
| < 45 (37) | 37 (9.5%) | Reference |  |
| 45-59 (95) | 95 (24%) | 0.52 (0.22, 1.22) | 0.13 |
| 60-74 (203) | 203 (52%) | 0.54 (0.25, 1.17) | 0.12 |
| ≥75 (54) | 54 (14%) | 0.47 (0.18, 1.25) | 0.13 |
| Polpy history |  |  |  |
| No (311) | 311 (80%) | Reference |  |
| Yes (78) | 78 (20%) | 2.23 (1.28, 3.87) | 0.004 |
| BMI (Kg/m^2^, continuous) | 23 ± 2.7 | 0.92 (0.84, 1.00) | 0.06 |
| Diabetes |  |  |  |
| No (318) | 318 (79%) | Reference |  |
| Yes (71) | 71 (18%) | 0.77 (0.42, 1.42) | 0.40 |
| Hypertension |  |  |  |
| No (185) | 185 (48%) | Reference |  |
| Yes (204) | 204 (52%) | 0.76 (0.47, 1.23) | 0.26 |
| Current smoke |  |  |  |
| No (339) | 339 (87%) | Reference |  |
| Yes (50) | 50 (13%) | 0.46 (0.19, 1.13) | 0.09 |
| Alcohol |  |  |  |
| No (353) | 353 (91%) | Reference |  |
| Yes (36) | 36 (9.3%) | 0.57 (0.21, 1.51) | 0.26 |
| Aspirins |  |  |  |
| No (343) | 343 (88%) | Reference |  |
| Yes (46) | 46 (12%) | 1.18 (0.57, 2.44) | 0.65 |
| Statins |  |  |  |
| No (344) | 344 (89%) | Reference |  |
| Yes (45) | 45 (12%) | 0.33 (0.11, 0.94) | 0.04 |
| ACEI |  |  |  |
| No | 375 (96%) | Reference |  |
| Yes | 14 (3.6%) | 3.93 (1.34, 11.56) | 0.01 |
| Vitamins |  |  |  |
| VB1(nmol/L) | 59 ± 9 | 1.03 (1.00, 1.06) | 0.03 |
| VB2 (ng/ml) | 241 ± 41 | 1.01 (1.00, 1.02) | 0.0008 |
| VB6 (μmol/ml) | 20 ± 5.0 | 1.06 (1.01, 1.10) | 0.02 |
| VB9 (nmol/L) | 14 ± 5.6 | 0.96 (0.91, 1.01) | 0.08 |
| VB12 (pg/ml) | 272 ± 99 | 1.00 (0.99, 1.00) | 0.02 |
| VA (μmol/ml) | 0.81 ± 0.24 | 1.33 (0.49, 3.57) | 0.58 |
| VC (μmol/ml) | 40 ± 5.5 | 1.05 (1.01, 1.10) | 0.01 |
| VE (ug/ml) | 11 ± 1.1 | 1.08 (0.87, 1.35) | 0.47 |

Supplementary Table. 5 The association between 25(OH)D per 10 nmol/L and CRC according to baseline characteristics.

| Sub-group | N | OR | 95% CI | P value | p value for interaction |
| --- | --- | --- | --- | --- | --- |
| Sex |  |  |  |  | 0.73 |
| Female | 158 | 0.98 | 0.95, 1.01 | 0.22 |  |
| Male | 231 | 0.97 | 0.96, 0.99 | 0.002 |  |
| Pooled | 389 | 0.97 | 0.96, 0.99 | 0.001 |  |
| Age, y |  |  |  |  | 0.21 |
| <45 | 37 | 0.97 | 0.91, 1.02 | 0.24 |  |
| 45-59 | 95 | 1 | 0.97, 1.03 | 0.81 |  |
| 60-74 | 203 | 0.98 | 0.96, 1.00 | 0.09 |  |
| ≥75 | 54 | 0.94 | 0.89, 0.99 | 0.02 |  |
| Pooled | 389 | 0.98 | 0.96, 0.99 | 0.007 |  |
| polyp history |  |  |  |  | 0.96 |
| No | 311 | 0.97 | 0.96, 0.99 | 0.007 |  |
| Yes | 78 | 0.98 | 0.95, 1.00 | 0.09 |  |
| Pooled | 389 | 0.98 | 0.96, 0.99 | 0.002 |  |
| BMI, Kg/m2 |  |  |  |  | 0.91 |
| < 18.5 | 51 | 0.97 | 0.94, 1.01 | 0.12 |  |
| 18.5-24.9 | 152 | 0.98 | 0.96, 1.00 | 0.09 |  |
| 25-30 | 186 | 0.98 | 0.96, 1.01 | 0.16 |  |
| Pooled | 389 | 0.98 | 0.97, 0.99 | 0.008 |  |
| Diabetes |  |  |  |  | 0.73 |
| No | 318 | 0.98 | 0.96, 0.99 | 0.006 |  |
| Yes | 71 | 0.99 | 0.95, 1.03 | 0.48 |  |
| Pooled | 389 | 0.98 | 0.96, 0.99 | 0.005 |  |
| Hypertension |  |  |  |  | 0.45 |
| No | 185 | 0.97 | 0.95, 0.99 | 0.01 |  |
| Yes | 204 | 0.98 | 0.96, 1.00 | 0.13 |  |
| Pooled | 389 | 0.98 | 0.96, 0.99 | 0.003 |  |
| Current smoke |  |  |  |  | 0.63 |
| No | 339 | 0.98 | 0.97, 1.00 | 0.02 |  |
| Yes | 50 | 0.97 | 0.92, 1.02 | 0.23 |  |
| Pooled | 389 | 0.98 | 0.97, 0.99 | 0.008 |  |
| Alcohol |  |  |  |  | 0.12 |
| No | 353 | 0.98 | 0.97, 1.00 | 0.02 |  |
| Yes | 36 | 0.92 | 0.85, 1.01 | 0.08 |  |
| Pooled | 389 | 0.98 | 0.96, 0.99 | 0.007 |  |
| Aspirin | |  |  |  | 0.0003 |
| No | 343 | 0.99 | 0.97, 1.00 | 0.10 |  |
| Yes | 46 | 0.87 | 0.8, 0.95 | 0.002 |  |
| Pooled | 389 | 0.98 | 0.96, 0.99 | 0.006 |  |
| Statins |  |  |  |  | 0.13 |
| No | 344 | 0.98 | 0.96, 0.99 | 0.002 |  |
| Yes | 45 | 1.02 | 0.97, 1.08 | 0.44 |  |
| Pooled | 389 | 0.98 | 0.96, 0.99 | 0.004 |  |

Abbreviations: BMI, body mass index.


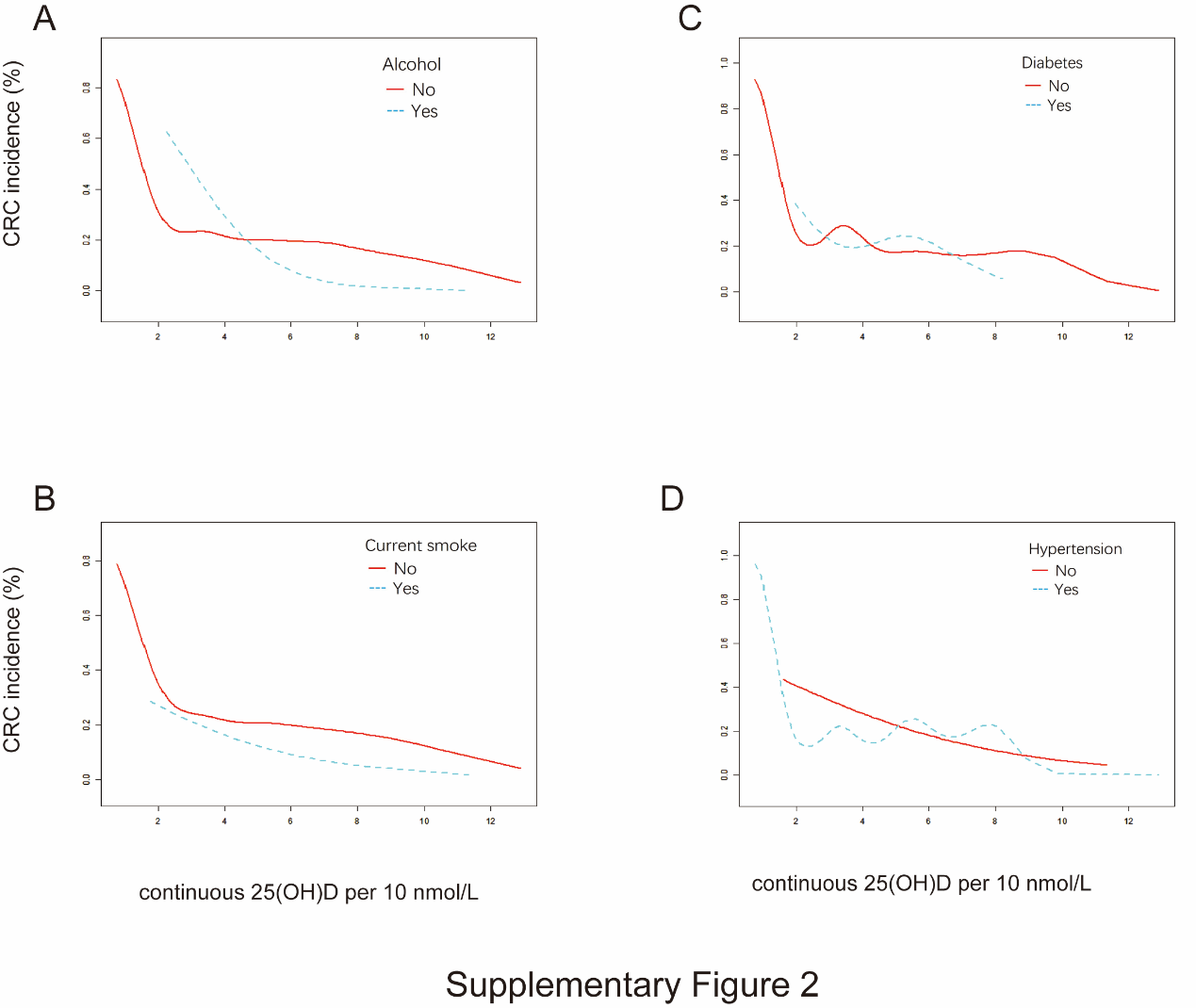


Supplementary figure.2 The association between continuous 25(OH)D per 10nmol/L and CRC according to baseline characteristics. Smooth fitting curve adjusted for sex, age, polyp history, diabetes, hypertension, smoke, alcohol, aspirin, Statins, ACEI, VA, VB1, VB6, VB9, VB12, VC, VE, VD and BMI. A: Auqa dashed line represents the spline plots of alcohol consumption, red solid represents no alcohol intake; B: Auqa dashed line represents the spline plots of smoke, red solid represents participants no smoke; C: Auqa dashed line represents the spline plots of diabetes, red solid represents participants without diabetes; D: Auqa dashed line represents the spline plots of hypertension, red solid represents participants without hypertension.
